# The Immunosuppressive Functions of Eosinophils Are Compromised in Patients With Allergic Rhinitis, Particularly Concerning Rab27a Expression

**DOI:** 10.1002/iid3.70091

**Published:** 2024-12-16

**Authors:** Yun Liao, Minyao Li, Shuo Song, Xuejie Xu, Xiaojun Xiao, Yu Liu, Gui Yang, Pingchang Yang

**Affiliations:** ^1^ Department of Otolaryngology Longgang Central Hospital Shenzhen China; ^2^ Department of General Practice Medicine Third Affiliated Hospital of Shenzhen University Shenzhen China; ^3^ Institute of Allergy & Immunology of Shenzhen University, State Key Laboratory of Respiratory Diseases Allergy Division at Shenzhen University and Shenzhen Key Laboratory of Allergy & Immunology Shenzhen China

**Keywords:** allergy, cytokine, eosinophil, immune regulation, rhinitis

## Abstract

**Background:**

Eosinophils have been acknowledged to be involved in the induction of numerous inflammatory disorders. There is still a lack of knowledge about whether eosinophils play a role in immune regulation. The aim of this study is to uncover the immune regulatory functions of eosinophils.

**Methods:**

Blood samples were collected from patients with allergic rhinitis (AR) and healthy control subjects. Peripheral blood mononuclear cells (PBMCs) were isolated from blood samples. Eosinophils were purified from PBMCs using flow cytometry cell sorting and analyzed using immunological approaches.

**Results:**

The results showed that eosinophils from healthy subjects had immune regulatory functions on T cell proliferation and cytokine release. Impairment of eosinophil immune regulatory functions was found in AR patients, which was associated with AR responses. Elevated Rab27a expression in eosinophils was associated with their impaired immune regulatory functions and the increased AR responses. Rab27a controlled the release of mediators from eosinophils. Low concentrations of Eosinophil mediators could trigger immune regulatory responses, while high concentrations could trigger inflammatory responses. Regulating Rab27a restored the immune regulatory functions of eosinophils of AR patients.

**Conclusions:**

Eosinophils have immune regulatory functions, which are controlled by the expression of Rab27a. Regulation of Rab27a can improve the immune regulatory functions of eosinophils. The data suggest that inhibition of Rab27a can be a drug candidate for the treatment of eosinophil‐related disorders.

## Graphical abstract

We found that human eosinophils had immune regulatory functions on suppressing the cell proliferation and cytokine release by other immune cells. The immune‐suppressive functions were impaired in eosinophils obtained from patients with allergic rhinitis.

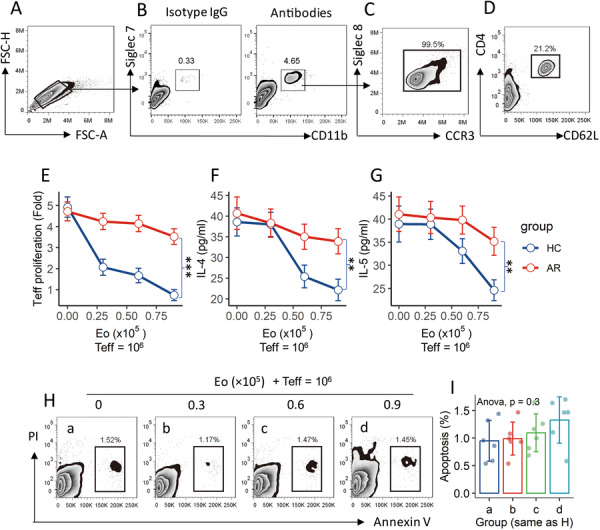

**Abbreviations**
Ab: antibody
AR: allergic rhinitis
CFSE: carboxyfluorescein succinimidyl este
DME: dust mite extracts
ECP: eosinophil cationic protein
EDN: eosinophil‐derived neurotoxic protein
EPX: eosinophil peroxidase
FCM: flow cytometry
HC: Healthy control
MBP: major basic protein
NS: nasal secretions
PBMC: peripheral blood mononuclear cell
PI: propidium iodide
PMA: phorbol myristate acetate
RE: relative expression
sIgE: specific IgE
SPT: skin prick test
Teffs: effector T cells

## 1 Introduction

The prevalence of allergic rhinitis (AR) is more than 10% in the world [1, 2]. The clinical symptoms of AR include paroxysmal attacks of sneezing, profound nasal discharge, and nasal obstruction. AR may complicate nasal polyposis, chronic rhinosinusitis, or even allergic asthma. The diagnosis of AR includes the AR history, positive skin prick test, and positive specific IgE in the serum [1]. The treatment of AR mainly focuses on controlling clinical symptoms, such as using steroid nasal spray and histamine antagonists. Specific allergen immunotherapy is an etiology‐targeting remedy for AR [3]. However, the pathogenesis of AR is not fully understood yet.

Eosinophils are one of the major effector cell fractions in AR, which are a fraction of immune cells. The white blood cells contain 1%–3% eosinophils, which can increase many inflammatory diseases, such as allergies [4]. Eosinophils may infiltrate into the local tissues during inflammation. A large number of granules are present in the cytoplasm of the cells. The contents of eosinophil granules, such as major basic protein (MBP), eosinophil cationic protein (ECP), eosinophil peroxidase (EPX), and eosinophil‐derived neurotoxic protein (EDN), are toxic, and are regarded as inflammation inducers [5]. Rab27a is essential in controlling eosinophil degranulation as reported [6]. The process of degranulation and exocytosis is heavily influenced by Rab27a, a small GTPase expressed by many cell types [7]. However, whether eosinophils contribute to immune regulation remains to be investigated. Whether the activity of Rab27a is involved in the regulation of eosinophil's immune regulatory function is unknown.

Immune regulatory cytokines are responsible for the majority of immune regulatory activities. There are several cytokines, such as IL‐10 and transforming growth factor (TGF)‐β, which have been recognized as playing crucial roles in immune regulatory activities [8, 9, 10]. These immune regulatory cytokines can suppress the activities of other immune cells, such as cell proliferation and cytokine release [8, 9, 10]. The mediators that eosinophils release have the potential to influence other immune cells. According to previous reports, eosinophils play a role in maintaining the body's homeostasis [11, 12]. Thus, we hypothesize that eosinophils have immune regulatory functions by releasing mediators in proper amounts. In the present study, we found that eosinophil mediators could suppress other immune cell activities. The expression of Rab27a played a critical role in governing the mediator release from eosinophils to render the induction of immune regulation or inflammation.

## 2 Materials and Methods

### 2.1 Reagents

Antibodies (Abs) of CD3 [Cat#: sc‐20047; fluorochrome: Alexa Fluor (AF)488], CD4 (sc‐19641, AF546), CD62L (sc‐390756, AF594), Siglec 7 (sc‐398919, AF648), CD11b (sc‐1186, AF687), CD14 (sc‐52457, AF700), and Rab27a (sc‐74586) were purchased from Santa Cruz Biotech (Santa Cruz, CA). ELISA kits of Rab27a, MBP, EPX, ECP, EDN, tryptase, IL‐4, IL‐5, IL‐13, IgE, IgG1, IL‐6, TNF‐α, and IFN‐γ were purchased from Dakewe BioMart (Shenzhen, China). Invitrogen (Carlsbad, CA) provided the reagents and materials for RT‐qPCR and Western blot analysis. PMA, LPS, ionomycin, and nexinhib20 were purchased from Sigma‐Aldrich (St. Louis, MO).

### 2.2 Human Subjects

At our allergy clinic, this study enrolled patients with perennial allergic rhinitis (AR). Our physicians followed our routine procedures for diagnosing AR, which can be found elsewhere. The diagnostic criteria include the following: patients had an AR history for more than 2 years. The skin prick test (SPT) and serum‐specific IgE showed positive results. The procedures of SPT and IgE assessment were reported previously [13, 14]. Patients with rhinosinusitis, nasal polyposis, other immune diseases, cancer, and severe organ diseases were excluded. Healthy control (HC) subjects were also included in the enrollment. The results of SPT and serum IgE were negative for all HC subjects. Our hospital's human ethics committee approved the use of human tissues in this study (Approval#: H2022003). Each human subject was asked to provide written informed consent. Table 1 presents the demographic data of human subjects.

**Table 1:** Demographic data of AR patients.

Items	AR patients	HC subjects
Number	30	30
Age (years)	33.2 ± 4.5	31.6 ± 3.8
Male (%)	15 (50)	15 (50)
Female (%)	15 (50)	15 (50)
FEV1 (% predicted)	100.2 (87.3, 102.2)	100.6 (88.1, 103.5)
Co‐suffer allergy		
Allergic asthma	3 (10%)	
Allergic dermatitis	1 (3.33%)	
Food allergy	1 (3.33%)	
Using corticosteroids^a^	30	
Blood neutrophil (10^9^/L)	5.11 (4.18, 6.44)	
Blood eosinophil (10^9^/L)	0.35 (0.16, 0.65)	
SPT results		
Mite mix	30 (100%)	
Timothy grass	1 (3.33%)	
Bermuda grass	1 (3.33%)	
Pine	1 (3.33%)	
Mold mix	6 (20%)	
Poplar	1 (3.33%)	
Rye	1 (3.33%)	
Mugwort	3 (10%)	
Animal dander	3 (10%)	

The data are presented as means ± SD or median (IQR).

Abbreviations: FEV1, forced expiratory volume in 1 s; SPT, skin prick test.

^a^ Patients used corticosteroid spray to control AR attacks.

### 2.3 Preparation of Peripheral Blood Mononuclear Cells (PBMCs)

An ulnar vein puncture was used to collect blood samples from human subjects. Gradient density centrifugation was used to isolate PBMCs from blood. The cells were used for further experiments.

### 2.4 Cell Culture

RPMI‐1640 medium was used to culture cells. Fetal calf serum (10%), penicillin (100 U/mL), streptomycin (0.1 mg/mL), and l‐glutamine (2 mM) were added to the medium. Cell viability was assessed by a Trypan blue exclusion assay, which was between 97% and 99%.

### 2.5 Flow Cytometry

Following published procedures [13, 14], cells collected from relevant experiments were stained with fluorescence‐labeled Abs or isotype IgG (Ab types are detailed in figures) with surface staining or/and intracellular staining. Cells were analyzed using a flow cytometer (BD FACSCanto II). The data were processed using the software package Flowjo with the data obtained from isotype IgG staining as a gating reference.

### 2.6 Isolation of Eosinophils, Monocytes and CD4^+^CD62L^+^ T Cells by Flow Cytometry (FCM) Cell Sorting

PBMCs were incubated with Abs labeled with fluorescence (diluted to 1 μg/mL, including CD3, CD4, CD62L, CD14, Siglec 7, and CD11b) for 30 min at 4°C. Cells were washed with FCM buffer (phosphate‐buffered saline, PBS, containing 2% bovine serum albumin, BSA) three times, and were analyzed using a flow cytometer (BD Aria). The CD3^+^ T cells were gated first, from which CD4^+^CD62L^+^ T cells were sorted to be used as effector T cells (Teffs). CD11b^+^Siglec 7^+^ cells were isolated to be eosinophils. CD14^+^ cells were isolated and used as monocytes. Cell purity was checked by FCM. If purity did not reach 90%, cells were sorted again.

### 2.7 Assessment of Apoptotic Cells

Cells (10^6^ cells/sample) obtained from relevant experiments were stained with propidium iodide (PI) and an Annexin V kit (Sigma‐Aldrich) following the protocol provided by the manufacturer. The cells were analyzed by FCM using the non‐staining cells as a negative control. The data were processed by Flowjo with the negative control sample as a gating reference. The Annexin V^+^ cells or/and PI^+^Annexin V^+^ cells were regarded as apoptotic cells.

### 2.8 Assessment of Teff Proliferation

Teffs were labeled with CFSE (carboxyfluorescein succinimidyl ester) and activated by exposing to PMA (50 ng/mL) and ionomycin (100 ng/mL) in culture for 3 days with or without the presence of eosinophils. The CFSE^+^ cells were counted by FCM, and presented as fold change.

### 2.9 ELISA Assay

The amounts of specific IgE (sIgE) and sIgG1 against dust mite extracts (DME) in nasal secretions (NS) were measured using an in‐house developed ELISA assay. Plate wells were coated with 2 µg/mL of DME, then blocked with blocking buffer (PBS containing 3% BSA), and incubated with NS samples (diluted to 1:10) in triplicate. A standard curve for quantifying IgG1 and IgE in NS samples was created by preparing 7 serial dilutions of human IgG1 or IgE on each plate. Bound IgG1 and IgE were detected by subsequent incubation with a biotinylated mouse anti‐human IgG1 and anti‐IgE, followed by a goat‐anti‐mouse Ab labeled with horseradish peroxidase. The assay was developed by addition of TMB and stopped with 2 M H_2_SO_4_. Absorbance (OD at 450 nm) was measured using a microplate reader (YT‐MB96A, Weifang, China).

### 2.10 Real‐Time Quantitative RT‐PCR (RT‐qPCR)

Eosinophils were prepared from blood samples collected from human subjects as described above. Following the manufacturer's instructions, a reverse transcription kit was used to convert RNA samples extracted from eosinophils into cDNA. Amplifying the cDNA samples was done using a Bio‐Rad CFX96 qPCR device and a SYBR Green Master Mix kit according to the manufacturer's instructions. The primers used in the study include *RAB27A* (tgggagactctggtgtaggg, ccctgctgtgtcccataact). The results were processed using the 2^‐∆∆Ct^ method and presented as relative expression (RE) against the *ACTB* gene (ggacttcgagcaagagatgg and agcactgtgttggcgtacag).

### 2.11 Determining Eosinophil Mediators Induce Inflammatory or Immune Regulatory Responses

Monocytes (10^6^ cells/mL) were cultured in the presence of MBP (a representative mediator of eosinophils) at gradient concentrations (detailed in figures) for 24 h. Supernatants were collected. The amounts of IL‐6 and TNF‐α (the representative inflammatory cytokines) in supernatants were determined by ELISA.

### 2.12 Assessment of the Role of Regulating Rab27a in Restoring the Immune Regulatory Functions of Eosinophils in AR Patients

Eosinophils and Teffs (labeled with CFSE) were cocultured at a ratio of 0.9 × 10^5^:1 × 10^6^ in the presence of PMA (50 ng/mL), ionomycin (100 ng/mL), and nexinhib20 at gradient concentrations (detailed in figures) for 2 days. Teff proliferation was assessed by FCM, the CFSE‐dilution assay. The amounts of IL‐4 and IFN‐γ in the supernatant were determined by ELISA with relevant reagent kits following the manufacturer's instructions.

### 2.13 Statistics

Student's *t*‐test or Mann–Whitney test were used to determine the difference between the two groups. More than two groups were subjected to ANOVA followed by the Bonferroni test. Spearman or Pearson correlation coefficient test was performed to determine the correlation between groups. The significant criterion was *p* < 0.05.

## 3 Results

### 3.1 Eosinophils From HC Subjects Exhibit Immune Regulatory Functions

**Figure 1:**
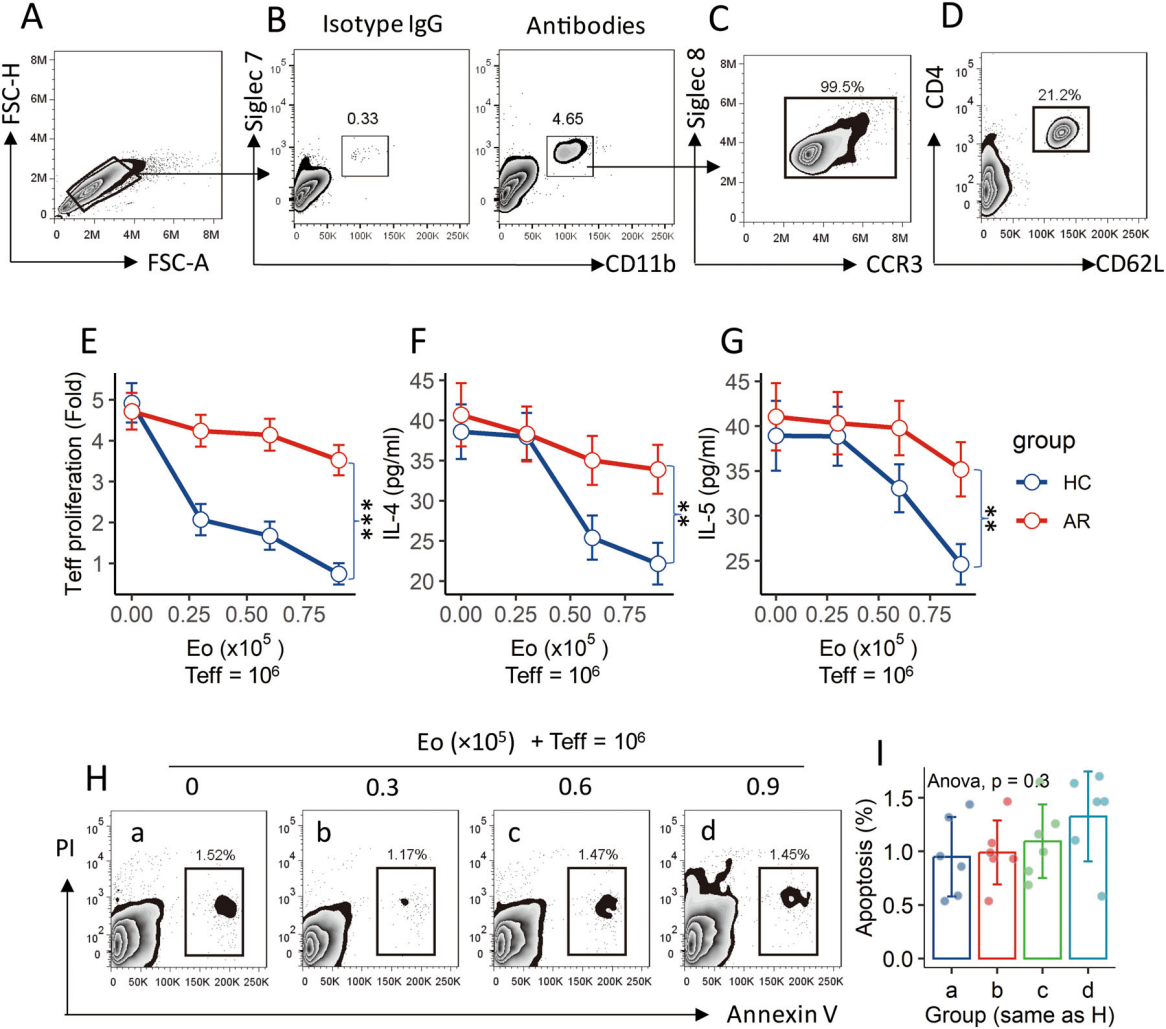
Assessment of eosinophil's immune‐suppressive ability. Eosinophils and CD4^+^CD62L^+^ T cells (Teffs) were isolated from PBMCs of HC subjects (*n* = 30) and AR patients (*n* = 30) (A–D). Eosinophils and Teffs were cocultured at gradient ratios (denoted on the X‐axis) in the presence of P&I for 2 days. (E–G) line plots show Teff proliferation (E; counted by FCM), the amounts of IL‐4 (F) and IFN‐γ (G) in culture supernatant. (H, I) Gated FCM plots show apoptotic cells. (I) Bars show mean ± SD of apoptotic cell counts from 6 experiments. Statistics: ANOVA. ***p < 0.001. AR, allergic rhinitis; FCM, flow cytometry; HC, healthy control; PBMC, peripheral blood mononuclear cell; P&I: PMA (phorbol myristate acetate; 50 ng/mL) and ionomycin (100 ng/mL) in culture; Teff, effector T cell.

Peripheral blood mononuclear cells (PBMCs) were prepared with blood samples collected from HC subjects. eosinophils and CD4^+^CD62L^+^ T cells (Effector T cells; Teffs) were isolated from PBMCs by flow cytometry (FCM) cell sorting (Figure 1A–D). Exposure to cell activators (phorbol myristate acetate [PMA] and ionomycin) induced Teff proliferation (Figure 1E), and release cytokines (IL‐4 and IFN‐γ) (Figure 1F‐G). The presence of eosinophils suppressed the activation of Teffs in an eosinophil number‐dependent manner. AR patients' eosinophils showed a weak suppressive capacity (Figure 1E–G). The results demonstrate that eosinophils have immune regulatory functions, which are impaired in those from AR patients. In addition, the interaction between eosinophils and Teffs did not result in cell apoptosis (Figure 1H–I). The cell viability was between 96% and 98% as assessed by Trypan blue exclusion assay.

### 3.2 Impairment of Eosinophil Immune Regulatory Functions in AR Patients Is Associated With AR Responses

**Figure 2:**
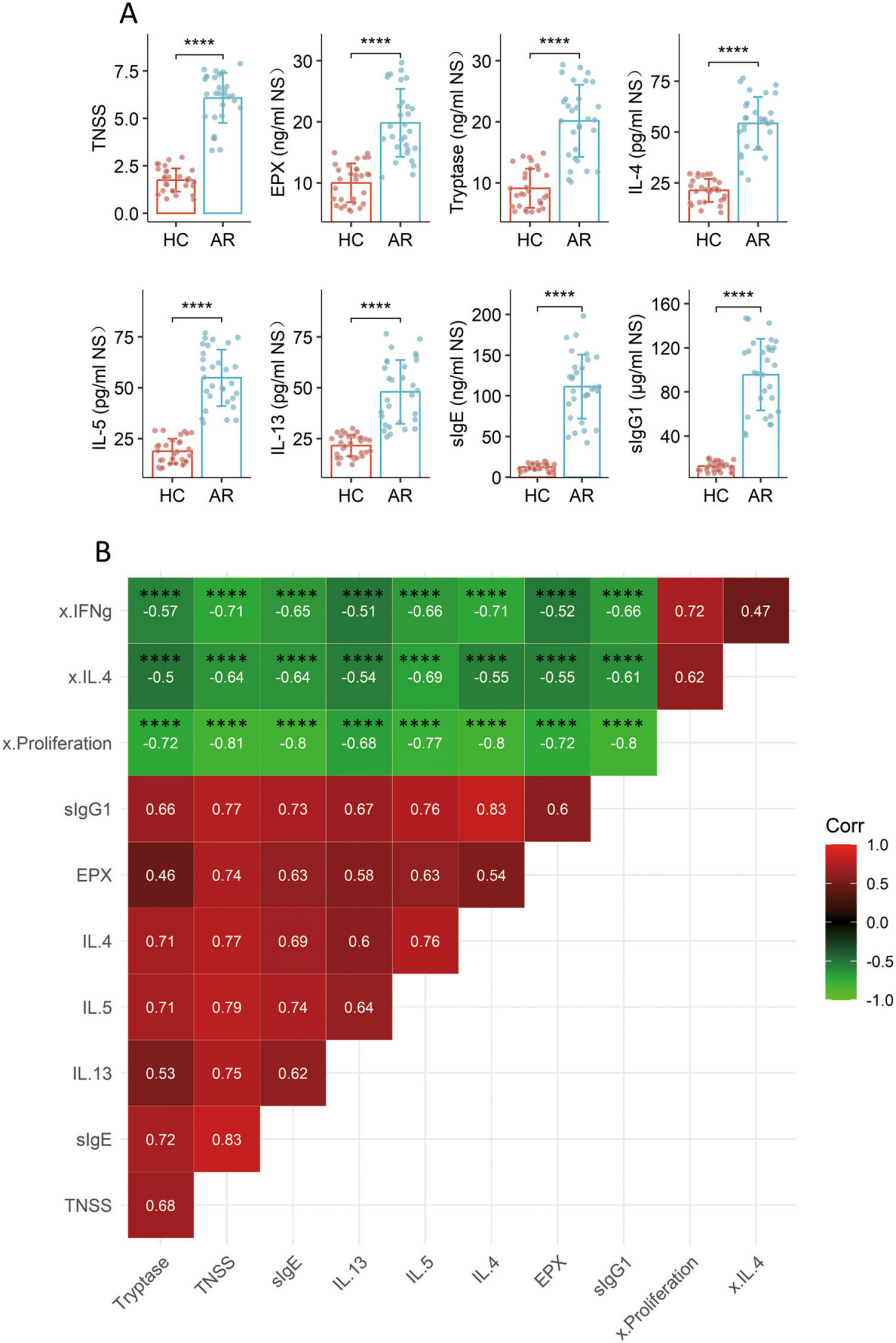
Correlation between the immune‐suppressive ability of eosinophils and AR responses. (A) The bars show mean ± SD of TNSS and the amounts of cytokines in NS. (B) The heatmap shows correlation coefficients between eosinophil immune‐suppressive ability (data are presented in Figure 1E‐G, the data of “0.9&10”) and the AR responses (the parameters in Figure 2A). Each dot in the bars presents one sample (tested in triplicate). Statistics: Mann–Whitney test (A) and Spearman correlation coefficient test (B). ****p < 0.0001. Abbreviations: AR, allergic rhinitis (*n* = 30); HC, healthy control (*n* = 30); NS, nasal secretion; TNSS, total nasal symptom scores; x, immune‐suppressive ability of eosinophils on the indicated cytokines.

The AR responses were recorded from AR patients, including the total nasal symptom scores, the amounts of EPX, tryptase, Th2 cytokines, specific IgE and IgG1 in nasal secretions, which were significantly higher in the AR group than that in the HC group (Figure 2A). The AR responses were negatively correlated with the immune‐suppressive functions of eosinophils (Figure 2B). The findings suggest that the pathogenesis of AR is linked to the impairment of immune regulatory functions of eosinophils.

### 3.3 The Expression of Rab27a in Eosinophils Is Associated With the Immune Regulatory Functions of Eosinophils and the AR Responses

**Figure 3:**
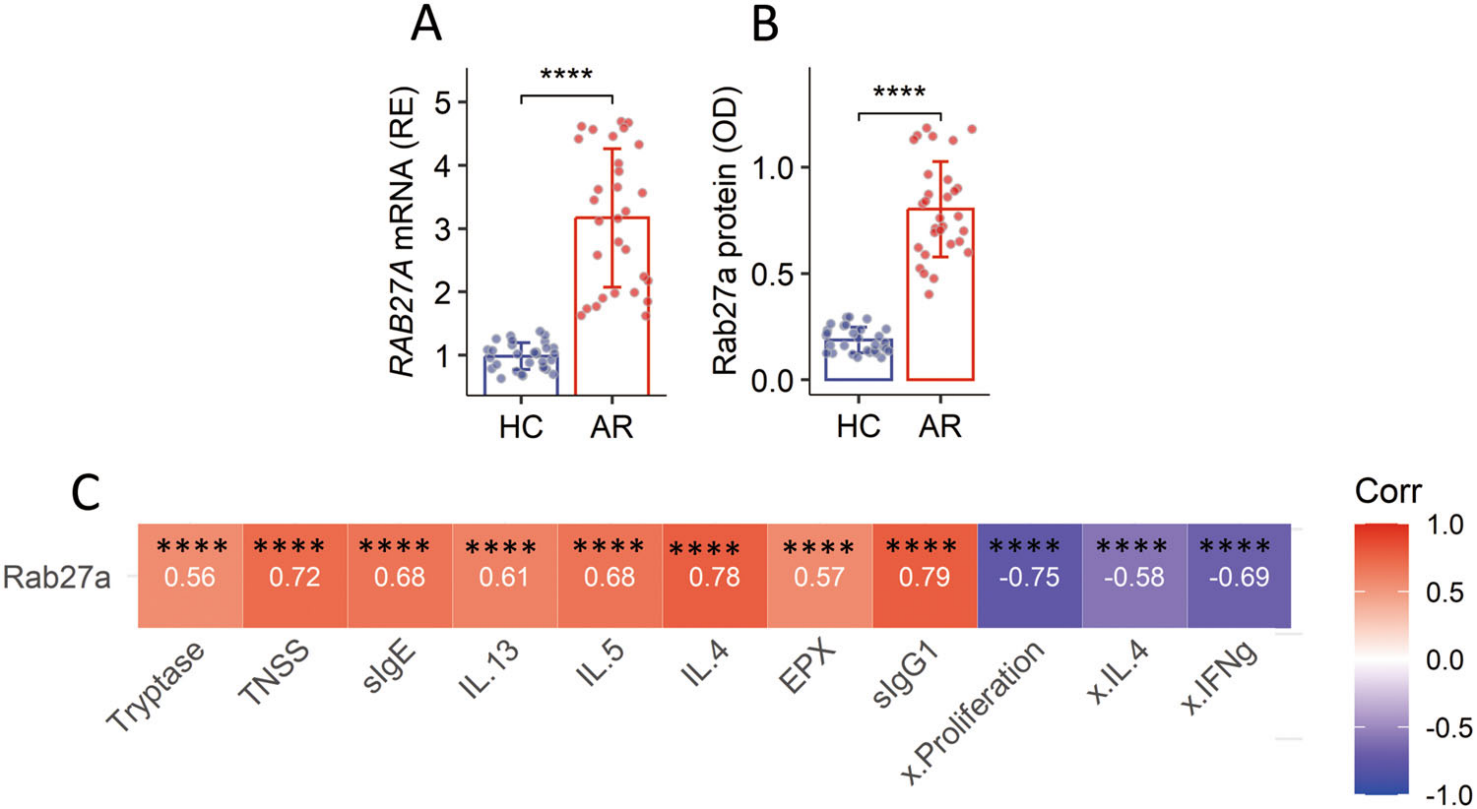
Assessment of Rab27a expression in eosinophils. Eosinophils were isolated from PBMCs, and analyzed by RT‐qPCR and Western blot analysis. (A) The amounts of Rab27a mRNA. (B) The amounts of Rab27a protein in cellular protein extracts of eosinophils (by ELISA). (C) Correlation coefficients between Rab27a mRNA and the parameters below heatmap. Each dot in bars presents one sample (tested in triplicate). Statistics: Mann–Whitney test (A), Student's *t*‐test (C), and Spearman correlation coefficient test (B). **p < 0.01; ****p < 0.0001. AR, allergic rhinitis (*n* = 30); HC, healthy control (*n* = 30); NS, nasal secretion; TNSS, total nasal symptom scores; x, immune‐suppressive ability of eosinophils on the indicated cytokines.

We then isolated eosinophils from PBMCs by FCM cell sorting. Eosinophils were analyzed by RT‐qPCR and Western blot analysis. The amount of Rab27a in the eosinophils of AR patients was higher than that in HC subjects (Figure 3A–C). The data of Rab27a mRNA and AR responses were found to have a positive correlation. A negative correlation was detected between the data of Rab27a mRNA and the immune‐suppressive functions of eosinophils (Figure 3D). The data indicate that the expression of Rab27a could be an important factor in controlling the immune regulatory functions of eosinophils.

### 3.4 Rab27a Governs the Release of Mediator From Eosinophils

**Figure 4:**
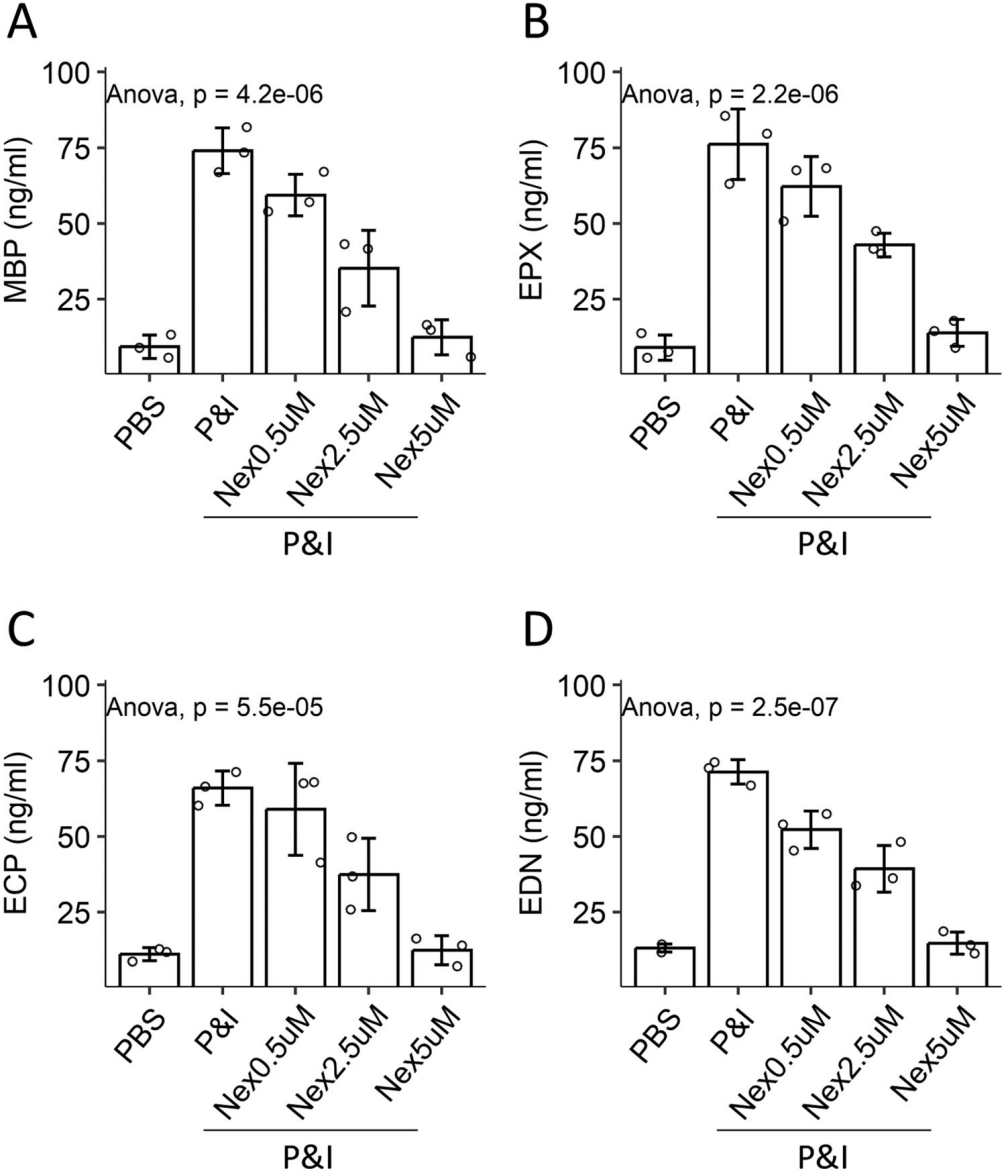
Assessment of the role of Rab27a in control the mediator release of eosinophils. Eosinophils were isolated from PBMCs of HC subjects, and cultured in the presence of reagents denoted on the *X*‐axis for 24 h. Supernatant was analyzed by ELISA. A–D. The bars show mean ± SD of indicated molecules in supernatant from three experiments. Each dot in the bars represents one sample (tested in triplicate). Statistics: ANOVA. HC, healthy control; P&I, PMA (50 ng/mL) and ionomycin (100 ng/mL); nex, Nexinhib20 (an inhibitor of Rab27a); PBMC, peripheral blood mononuclear cell.

Eosinophils were isolated from PBMCs by FCM cell sorting. To activate eosinophils, PMA/ionomycin was added to the culture medium. The amounts of eosinophil mediators (including MBP, ECP, EPX, and EDN) were increased in the culture supernatant, indicating that eosinophils release mediators upon activation. The addition of nexinhib20, an inhibitor of Rab27a, to the culture medium blocked the mediator release in a dose‐dependent manner (Figure 4). The results indicate that Rab27a controls the release of mediators by eosinophils.

### 3.5 Eosinophil Mediators Induce Immune Regulatory or Inflammatory Responses in a Dose‐Dependent Manner

**Figure 5:**
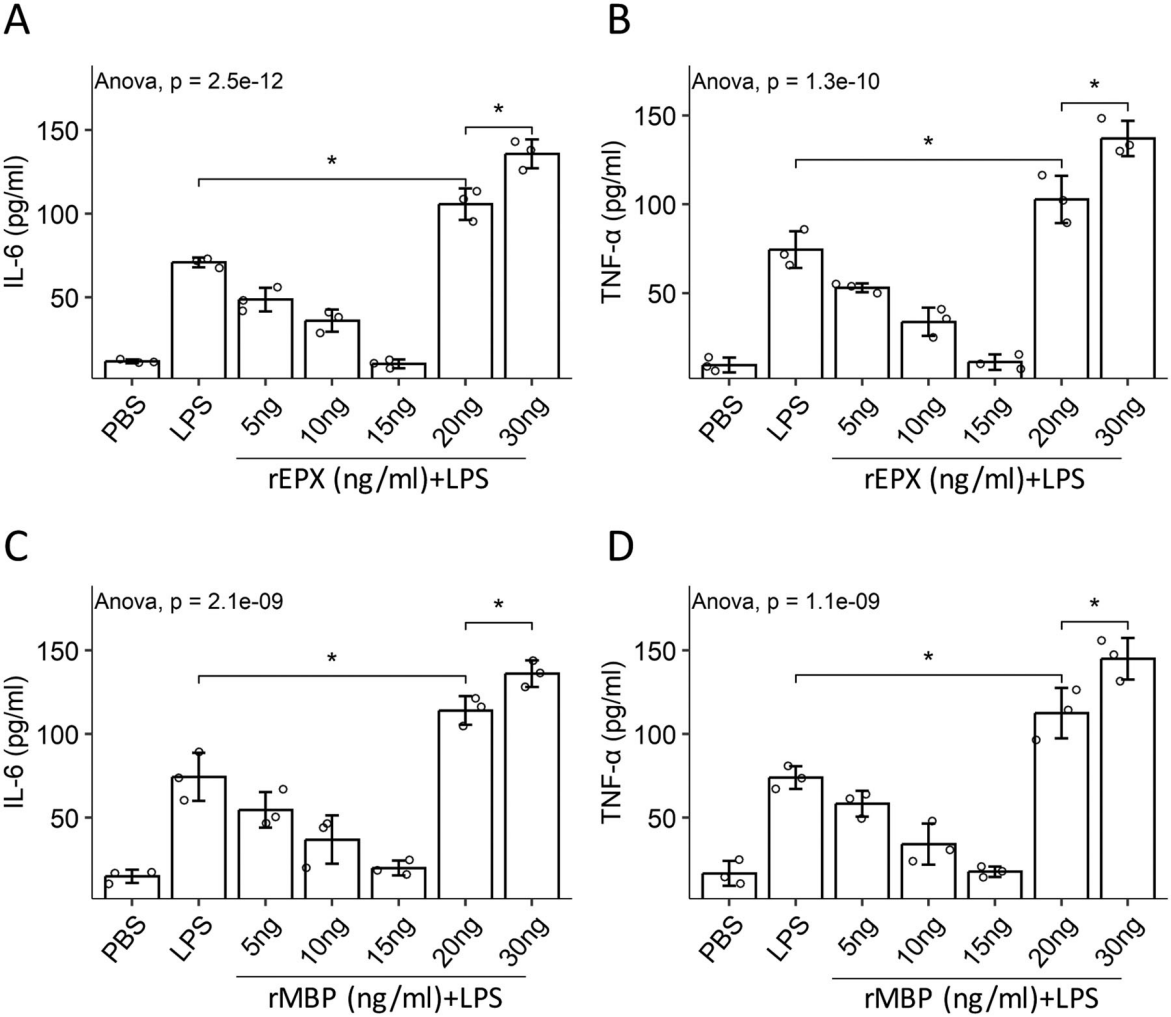
Eosinophil mediators inducing immune regulatory or inflammatory responses is dose‐dependent. Monocytes were isolated from PBMCs, and cultured in the presence of LPS (10 μg) + rEPX or LPS + rMBP at gradient concentrations for 24 h. A–D. Bars show mean ± SD of the amounts of indicated molecules in culture supernatant. Each dot in the bars represents one sample (tested in triplicate). Statistics: ANOVA + Bonferroni test. PBMC, peripheral blood mononuclear cell; rEPX and rMBP, recombinant EPX and MBP.

Monocytes (CD14^+^) were isolated from PBMCs by FCM cell sorting. Lipopolysaccharide (LPS) was added to the culture medium to activate monocytes. We found that LPS induced monocytes to release IL‐6 and TNF‐α (regarded as the representative inflammatory cytokines). The presence of eosinophil mediator, recombinant EPX or MBP, effectively suppressed the release of cytokines in the dosage range of 5–15 ng/mL, while the dosage of 20–30 ng/mL significantly enhanced the release of cytokines (Figure 5). The findings indicate that eosinophil mediators have immune‐suppressive abilities at low concentrations, but they promote inflammatory responses at high concentrations.

### 3.6 Regulating Rab27a Restores the Immune Regulatory Functions of Eosinophils of AR Patients

**Figure 6:**
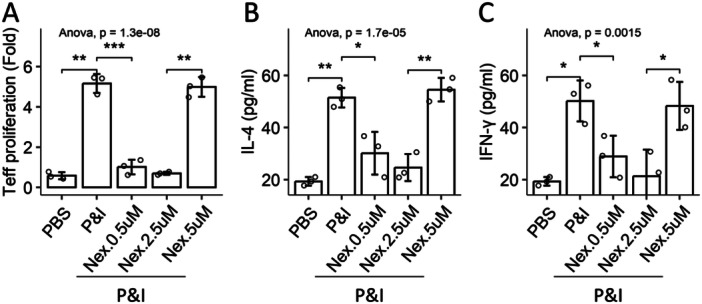
Inhibiting Rab27a restores AR eosinophils' immune‐suppressive ability. Eosinophils and CD4^+^ T cells (Teffs) were isolated from PBMCs of AR patients. Eosinophils and Teffs were cultured at a ratio of 0.9 × 10^5^:1 × 10^6^ in the presence of P&I and nexinhib20 at gradient concentrations for 2 days. Bars show mean ± SD of Teff proliferation (A) and the amounts of IL‐4 (B) and IFN‐γ (C) in the culture supernatant. Each dot in the bars represents one sample (tested in triplicate). Statistics: ANOVA + Bonferroni test. The experiments were repeated three times. Abbreviations: nex, Nexinhib20 (an inhibitor of Rab27a); PBMC, peripheral blood mononuclear cell. P&I, PMA (50 ng/mL) and ionomycin (100 ng/mL).

Eosinophils and CD4^+^ T cells were isolated from PBMCs. Gradient concentrations of Nexinhib20 were added to the culture medium of the coculture of eosinophils and CD4^+^ T cells. We found that exposure to PMA/ionomycin induced CD4^+^ T cell proliferation, and the release of IL‐4 and IFN‐γ. The presence of AR eosinophils did not show an effect on suppressing the activities of CD4^+^ T cells. The presence of nexinhib20 (an inhibitor of Rab27a) enhanced the immune‐suppressive functions of AR eosinophils in the dosage range from 0.5 to 2.5 μM. When the concentrations of nexinhib20 exceeded 5 μM, the immune‐suppressive functions of eosinophils were diminished (Figure 6A–C). The amounts of eosinophil mediators in culture supernatant were in parallel to the nexinhib20 dosage from 0.5 to 2.5 μM, which was reversed at the dosage of 5 μM (Figure 6, Table 2). The results indicate that regulating Rab27a in a proper range can restore the immune regulatory functions of the AR eosinophil.

**Table 2:** Comparison between eosinophil mediator release and inflammatory responses.

Nexinhib20	EPX	MBP	ECP	EDN	Proliferation	IL‐4	IFN‐γ
*Nex.0 μM*	74.02	74.02	65.94	71.28	4.4	48.36	45.83
Nex.0.5 μM	59.36	59.36	58.92	52.23	2.34	30.08	28.87
Nex.2.5 μM	35.19	35.19	37.39	39.25	0.7	24.62	21.29
Nex.5 μM	12.35	12.35	12.39	14.66	4.99	54.51	48.26

Abbreviation: Nex, Nexinhib20.

## 4 Discussion

The present data show that, besides being inflammatory inducers, eosinophils also have immune regulatory functions, which are impaired in AR patients. Eosinophil mediators in the right concentration range can have immune regulatory functions that suppress other immune cell activities. However, excessive levels of eosinophil mediators can induce inflammatory responses. Rab27a expression is crucial for the direction of eosinophils in causing immune regulation or inflammation.

Eosinophils are well known to play a crucial role in inducing inflammatory responses in the body. For example, eosinophils are the major inflammation inducers in eosinophil esophagitis, eosinophil gastritis, eosinophil ileitis, and eosinophil colitis in the gastrointestinal tract [15]. In the airway inflammatory disorders, eosinophils also play a crucial role. Such as allergic asthma, eosinophil pulmonary inflammation, and chronic obstructive pulmonary diseases [16]. However, the biological significance of eosinophils may not only involve inducing inflammation in the body. Others propose that eosinophils play an important role in maintaining the homeostasis in the intestine in response to microbe infection [17]. Eosinophils also play a critical role in tissue development, metabolic control, and remodeling [11, 12]. Our data show that eosinophils have immune regulatory functions. Eosinophil mediators are capable of effectively suppressing the activities of other immune cells. Eosinophils have the ability to trigger either inflammation or immune regulation depending on the specific micro‐environment.

We found that the immune regulatory functions of eosinophils were dependent on the eosinophil mediator. This was verified by using recombinant proteins of MBP and EPX. The rMBP or rEPX effectively suppressed T cell proliferation and cytokine release of T cells. Thus, the rationale for eosinophil's immune regulatory functions is similar to those released by regulatory T cells and regulatory B cells. Previous reports indicate that transforming growth factor (TGF)‐β can dampen other T cell proliferation and metabolism through the TGF‐β‐Smad3 signal pathway [18]. Regulatory B cells produce IL‐10, which restrains the T follicular helper cell activities in patients with primary Sjögren's syndrome [19]. Current data indicate that eosinophils have the ability to regulate other immune cells' activities, and can be one of the immune regulatory cell fractions.

It is widely agreed that the granule contents in the cytoplasm of eosinophils are cytotoxic and can induce inflammation. The presence of eosinophil granular substances, including MBP, EPX, ECP, and EDN, in the sputum of asthma patients, is believed to be a sign of the degree of their illness [20]. It is suggested that the measurements of EPX can be an important parameter in the diagnosis of eosinophil‐related diseases [21]. These data indicate that mediators released by eosinophils can influence the activities of other cells. Most studies on this topic record the effects of eosinophil‐derived mediators on the induction of inflammation. Current data show that eosinophil mediators present the immune regulatory functions at low concentrations, while the induction of inflammation is presented at high concentrations of eosinophil mediators. This is in line with the reality. Healthy people also have eosinophils, which do not induce inflammation in general. While eosinophils in patients with related diseases release a relatively large quantity of mediators, this can induce inflammation. This prompted us to conclude that there is a trigger in eosinophils that directs the activities of eosinophils toward immune regulation or inflammation.

It has been known that eosinophils express Rab27a [6, 22]. Our data are in line with those pioneer studies by showing that eosinophils express Rab27a, which is higher in those isolated from AR patients as compared to those of HC subjects. Rab27a is a small GTPase and is expressed by many cell types [7]. Rab27a plays a crucial role in the process of degranulation and exocytosis [7]. Rab27a promotes various steps in membrane traffic and can form complexes with target proteins. The complex is incorporated onto the surface of cells. Subsequently, the two proteins separate. The Rab27a can be recycled and re‐function, which is controlled by two regulatory enzymes, a GEF (guanine nucleotide exchange factor; the activator) and a GAP (GTPase‐activating protein; the inactivator) [23, 24]. Current information indicates that Rab27a levels in eosinophils of patients with AR are elevated. The amount of Rab27a in eosinophils has a positive correlation with the amount of eosinophil mediator release. The release of eosinophil mediators can be suppressed by inhibition of Rab27a. These data indicate that Rab27a plays a key role in governing eosinophil's mediator release. Rab27a is implicated in the cause of Griscelli syndrome type 2, a congenital error of immunity that results in partial albinism and hemophagocytic lymphohistiocytosis. The occurrence of this event is attributed to *RAB27A* mutations [25]. A scenario about this event is that when eosinophils express low amounts of Rab27a, the released small number of mediators render immune regulatory functions, while the high expression of Rab27a induces a large amount of mediator release to induce inflammatory responses.

To sum up, the findings show that eosinophils can trigger immune regulation or inflammatory responses depending on the amounts of mediators released. Rab27a governs the release of eosinophil mediators. Inhibition of Rab27a can reduce the mediator release of eosinophils, which has the translation potential to be developed into a drug candidate for the treatment of eosinophil‐related diseases.

Limitation of this study: The present data are generated from cells obtained from human subjects. Further studies using animal models are warranted to further elucidate the mechanism by which eosinophils fulfill their immune regulatory functions. Previous reports indicate induction of regulatory T cells (Treg cells) in vivo study, or adoptive transplantation of Treg cells can suppress ongoing immune‐inflammatory disorders [26, 27]. Whether modulation of eosinophils in vivo can modulate the inflammatory process is an interesting point and warranted to be investigated. Additionally, we only collected samples from Chinese people in the Mainland of China. Whether immigrants from China to other countries also present a similar phenomenon is an interesting topic and needs to be investigated further.

## Author Contributions

**Yun Liao:** data curation, investigation. **Minyao Li:** data curation, investigation. **Shuo Song:** data curation, investigation. **Xuejie Xu:** data curation, investigation. **Xiaojun Xiao:** data curation, investigation. **Yu Liu:** data curation, investigation. **Gui Yang:** data curation, investigation, superversion. **Pingchang Yang:** conceptualization, project administration, supervision, writing–original draft, writing–review and editing. All authors reviewed the manuscript.

## Conflicts of Interest

The authors declare no conflicts of interest.

## Data Availability

Data are available upon request. All the data are included in this paper.
